# Zonisamide enhances neurite outgrowth from adult rat dorsal root ganglion neurons, but not proliferation or migration of Schwann cells

**DOI:** 10.1007/s00418-019-01839-8

**Published:** 2019-12-26

**Authors:** Shizuka Takaku, Kazunori Sango

**Affiliations:** Diabetic Neuropathy Project, Department of Sensory and Motor Systems, Tokyo Metropolitan Institute of Medical Science, 2-1-6 Kamikitazawa, Setagaya-ku, Tokyo, 156-8506 Japan

**Keywords:** Zonisamide, Dorsal root ganglion neurons, Neurite outgrowth, ND7/23 cells, Signaling pathways, Immortalized Schwann cells

## Abstract

Zonisamide, an anti-epileptic and anti-Parkinson’s disease drug, displays neurotrophic activity on cultured motor neurons and facilitates axonal regeneration after peripheral nerve injury in mice, but its underlying mechanisms remain unclear. In this study, zonisamide enhanced neurite outgrowth from cultured adult rat dorsal root ganglion (DRG) neurons in a concentration-dependent manner (1 μM < 10 μM < 100 μM), and its activity was significantly attenuated by co-treatment with a phosphatidyl inositol-3′-phosphate-kinase (PI3K) inhibitor LY294002 or a mitogen-activated protein kinase (MAPK) inhibitor U0126. In agreement with these findings, 100 μM zonisamide for 1 h induced phosphorylation of AKT and ERK1/2, key molecules of PI3K and MAPK signaling pathways, respectively in mouse neuroblastoma × rat DRG neuron hybrid cells ND7/23. In contrast, zonisamide failed to promote proliferation or migration of immortalized Fischer rat Schwann cells 1 (IFRS1). These findings suggest that the beneficial effects of zonisamide on peripheral nerve regeneration may be attributable to its direct actions on neurons through PI3K and MAPK pathways, rather than the stimulation of Schwann cells.

## Introduction

Successful axonal regeneration in the peripheral nervous system (PNS) depends on the capacity of neurons and/or Schwann cells to regenerate neurites, the environment distal to the injury, and the target tissues receptive to reinnervation (Sango et al. 2017). The sequence of cellular events during Wallerian degeneration includes Schwann cell activation and proliferation, macrophage recruitment, elimination of axonal and myelin debris, and synthesis of neurotrophic and chemotactic factors. However, axonal regeneration and reinnervation depend on appropriate contact of regenerating axonal sprouts with Schwann cell basal laminae in the distal nerve segment. Clinical approaches to repair axonal injury are still far from satisfactory, and no effective drugs are available to promote axonal regeneration with functional restoration after injury (Faroni et al. 2015).

Drug repositioning or repurposing is a strategy to discover new efficacies of drugs currently applicable to patients, and may significantly diminish the cost and time for developing drugs as compared with conventional approaches (Padhy and Gupta 2011). Zonisamide, a benzisoxazole derivative, was initially developed as an anti-epileptic drug (Jain 2000), and has been successfully repositioned for Parkinson’s disease (Murata et al. 2015; Murata et al. 2016). Yagi et al. (2015) found that zonisamide promoted neurite outgrowth from cultured motor neurons and facilitated axonal regeneration after sciatic nerve injury in mice. These findings provide evidence of neurotrophic and neuroprotective properties of zonisamide in the PNS and suggest its potential repositioning for peripheral nerve injury; however, the underlying mechanisms remain unclear. By employing primary-cultured adult rodent sensory neurons of dorsal root ganglia (DRGs) and immortalized Fischer rat Schwann cells 1 (IFRS1) (Sango et al. 2011), we investigated the functional roles of numerous growth factors and cytokines in peripheral nerve degeneration and regeneration (Sango et al. 2008; Takaku et al. 2013; Tsukamoto et al. 2015a, b). The aim of the present study is to elucidate the potential efficacy of zonisamide for neurite outgrowth from DRG neurons and proliferation/migration of IFRS1. In addition, neuroblastoma × DRG neuron hybrid ND7/23 cells (Wood et al. 1990) were employed for investigating the signaling molecules and pathways mediating the neurite outgrowth-promoting effects of zonisamide.

## Materials and methods

### Primary culture of adult rat DRG neurons

Three-month-old female Wistar rats were obtained from Japan Clea (Shizuoka, Japan). All the experiments were conducted in accordance with the Guideline for the Care and Use of Animals (Tokyo Metropolitan Institute of Medical Science 2011). Dissociated cell culture of DRG neurons was performed as previously described (Takaku et al. 2013). Briefly, DRGs from the cervical to the lumbar level were dissected from each animal and dissociated with collagenase (CLS-3; Worthington Biochemicals, Freehold, NJ, USA) and trypsin (Sigma, St. Louis, MO, USA). These ganglia were subjected to density gradient centrifugation (5 min, 200*g*) with 30% Percoll PLUS™ (GE Healthcare Bio-Sciences Corp., Piscataway, NJ, USA) to eliminate the myelin sheath. This procedure resulted in a yield of more than 5 × 10^4^ neurons along with a smaller number of Schwann cells and fibroblasts.

### Neurite outgrowth assay

The dissociated DRG neurons were suspended in Dulbecco’s Modified Eagle’s medium (DMEM; Thermo Fisher Scientific Inc., Waltham, MA, USA) with 10% fetal bovine serum (FBS; Thermo Fisher) and seeded onto poly-L-lysine (PL; Sigma, 10 μg/mL)-coated wells of 8-well chamber slides (Nalge Nunc International, Naperville, IL, USA). The density of neurons was adjusted to approximately 2 × 10^3^/cm^2^ in each well. After remaining in the serum-containing medium for 16 h, the cells were cultured for 32 h in DMEM with serum-free medium supplement B27 (Thermo Fisher) and different concentrations (0, 1, 10, or 100 μM) of zonisamide. Then the neurons were fixed with acid alcohol (95% ethanol and 5% acetic acid) at 4 °C for 10 min, incubated overnight at 4 °C with the mouse anti-βIII tubulin monoclonal antibody (1:2000; Sigma) and then incubated for 1 h at 37 °C with the peroxidase-conjugated anti-mouse IgG antibody (1:200; MBL Corp., Ltd., Nagoya, Japan). Immunoreactions were visualized under a light microscope with 0.01% diaminobenzidine tetrahydrochloride (Wako Co., Tokyo, Japan) and 0.01% hydrogen peroxide (Sigma) in 50 mM Tris buffer (pH 7.4) at 37 °C for 10 min. The average neurite length (in μm) was calculated from the measurements of approximately 700 neurites from 5 different cultures of each experimental group using digital images (MetaMorph System, Molecular Devices, Inc., Sunnyvale, CA, USA), and was normalized as the relative value of the control group.

To investigate the signaling pathways mediating the promoting effects of zonisamide on neurite outgrowth, 10 μM of a phosphatidylinositol-3′-phosphate-kinase (PI3K) inhibitor LY294002 (Cell Signaling Technology, Beverly, MA, USA) or a mitogen-activated protein kinase (MAPK) kinase (MEK) inhibitor U0126 (Calbiochem; EMD Chemicals, Inc., San Diego, CA, USA) were co-treated with 100 μM zonisamide.

### Culture of ND7/23 cells and western blotting

Mouse neuroblastoma/rat embryonic DRG neuron hybrid ND7/23 cells (Wood et al. 1990) were kindly provided by Prof. Atsufumi Kawabata and Dr. Fumiko Sekiguchi, Laboratory of Pharmacology and Pathophysiology, Faculty of Pharmacy, Kindai University, Higashi-Osaka, Japan (Mitani et al. 2016). The cells at the passage of 15–20 were maintained in DMEM supplemented with 5% FBS, and employed for western blot analysis (Takaku et al. 2013). The cells at semi-confluency in 60 mm culture plates were incubated in DMEM/B27 with 100 μM zonisamide for 0, 5, 10, 30, 60 and 120 min, and lysed with 2 × sodium dodecyl sulfate (SDS) sample buffer. SDS–polyacrylamide gel electrophoresis (SDS-PAGE) was performed using 10% SDS-PAGE gel (Fujifilm, Tokyo, Japan). After electrophoresis, the protein was transferred onto a PVDF membrane with an electroblotter (Nihon Eido Co., Ltd., Tokyo, Japan). The membrane was incubated in Tris-buffered saline with 0.1% Tween 20 (including 5% skimmed milk or 5% bovine serum albumin) for 1 h at room temperature, and then overnight at 4 °C with the following antibodies:

1. anti-AKT antibody (1:1000; Cell Signaling);
2. anti-phospho-AKT antibody (1:1000; Cell Signaling);
3. anti-extracellular signal-regulated kinase (ERK) 1/2 antibody (1:1000; Cell Signaling);
4. anti-phospho-ERK1/2 (pT202/pY204) antibody (1:2000; Cell Signaling).
5. After rinsing with PBS containing 0.1% Tween 20, the membrane was incubated in a solution of HRP-conjugated anti-rabbit IgG antibody or anti-mouse IgG antibody (1:2000; MBL) for 1 h. After rinsing, immunocomplexes on the membrane were visualized with ECL plus a Western Lighting Ultra (PerkinElmer, Inc., Waltham, MA, USA).

### Culture of IFRS1 and assays for proliferation/survival and migration

Spontaneously immortalized Fischer rat Schwann cells 1 (IFRS1) from adult Fischer 344 rats (Sango et al. 2011) at the passage of 30–40 were maintained in DMEM supplemented with 5% FBS, and employed for the following assays.

The effects of zonisamide on the proliferation/survival of IFRS1 were evaluated using the CellTiter 96^®^ AQueous One Solution Cell Proliferation Assay kit (Promega, Madison, WI, USA) according to the manufacturer’s instructions (Tsukamoto et al. 2015b). The cells were seeded onto each well of 96-well culture plates at an approximate density of 3 × 10^4^/cm^2^, and were incubated overnight in DMEM supplemented with 5% FBS. The cells were then maintained in DMEM/1% FBS with different concentrations (0, 1, 10, or 100 μM) of zonisamide or 2 μM forskolin as a positive control (Yamada et al. 1995; Sango et al. 2011) for 1 day and 3 days. After rinsing with 250 μL of DMEM, the cells were incubated for 1 h at 37 °C in 100 μL of DMEM with 10 μL of CellTiter 96^®^ AQueous One Solution Reagent, and absorbance at 490 nm was determined with a microplate reader (Varioskan Flash; Thermo Fischer).

The effects of zonisamide on IFRS1 cell migration were evaluated by the scratch wound assay (Liang et al. 2007; Roloff et al. 2013). Briefly, the cells were seeded onto a PL-coated 35 mm glass-bottomed dish with grid (Matsunami Glass Ind., LTD, Osaka, Japan) at an approximate density of 2 × 10^4^/cm^2^, and maintained in DMEM/5%FBS for 24 h. Then a cell-free area was scratched using a sterile 200 μL pipette tip (BM Equipment Co., Ltd, Tokyo, Japan). The cells were incubated in DMEM/1%FBS in the presence or absence of 100 μM zonisamide, and the scratch was photographed 2 h (0 day) and 3 days after its generation using a phase-contrast microscope (IMT-2; Olympus, Tokyo, Japan) equipped with a microscope digital camera system (DP22-CU; Olympus) and image analysis software (WinROOF2015; Mitani Corporation, Tokyo, Japan). The number of cells migrating into the square area of scratch (6 × 6 grids) was calculated by reducing the number in the area at 3 days from that at 0 day, and expressed as the relative value of the control condition.

### Statistical analysis

All the data are expressed as means with SD, and the number of experiments is indicated in the figure legends. Parametric comparisons between experimental groups were performed by one-way analysis of variance (ANOVA); when ANOVA showed a significant difference between groups (*P* < 0.05), Tukey–Kramer test was used to identify which group differences accounted for the significant *P* value.

## Results and discussion

### Zonisamide promotes neurite outgrowth from DRG neurons

**Fig. 1:**
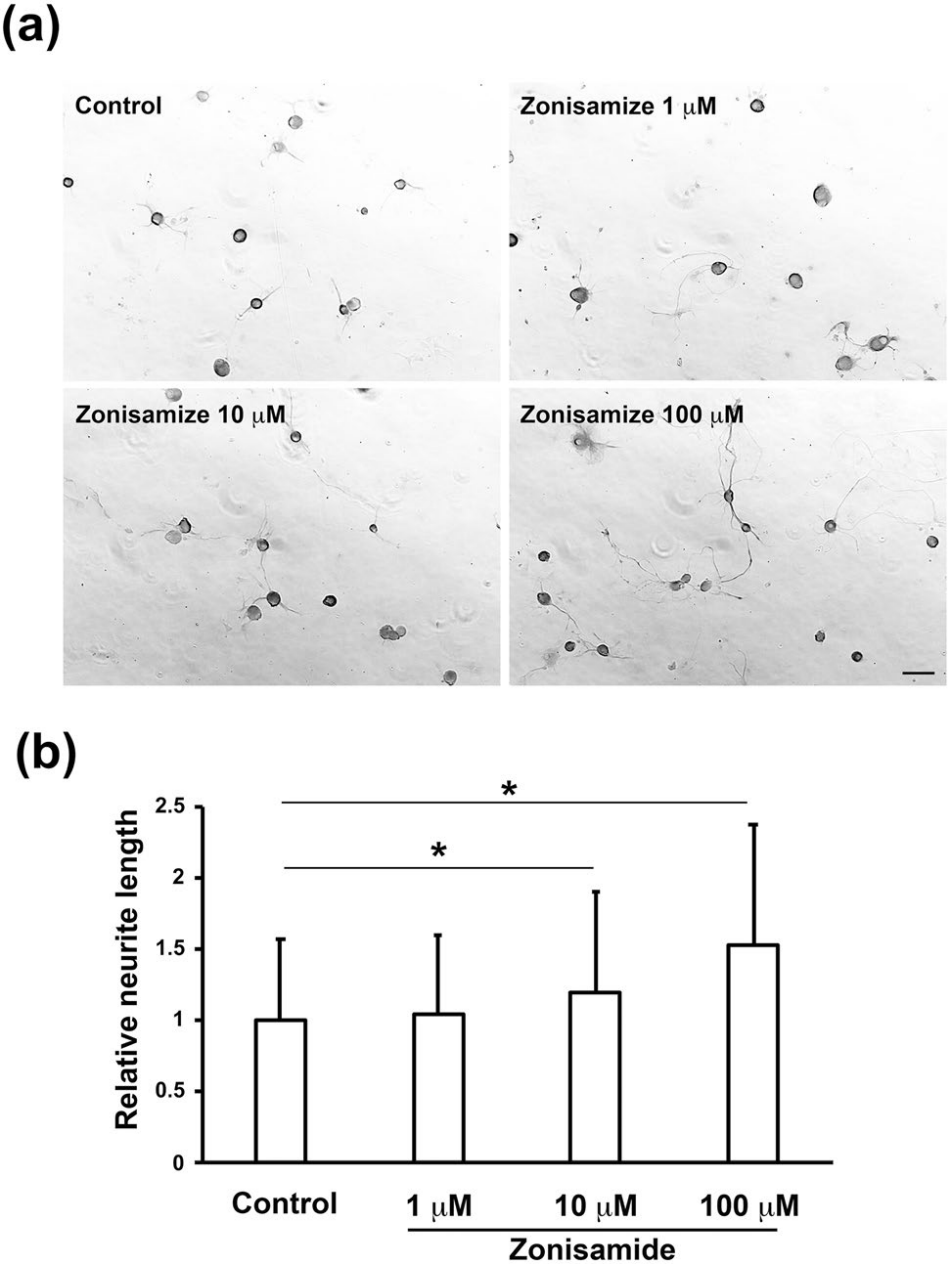
Zonisamide promotes neurite outgrowth from adult rat DRG neurons after 2 days in culture. **a** Representative photomicrographs of control and zonisamide (1 μM, 10 μM, and 100 μM)-treated DRG neurons immunostained with anti-βIII tubulin antibody. Scale bar = 50 μm. **b** Concentration-dependent effects of zonisamide on neurite outgrowth; bar charts of the relative neurite length in each culture condition. Values represent means + SD from 665 to 816 neurites; *P < 0.01

**Fig. 2:**
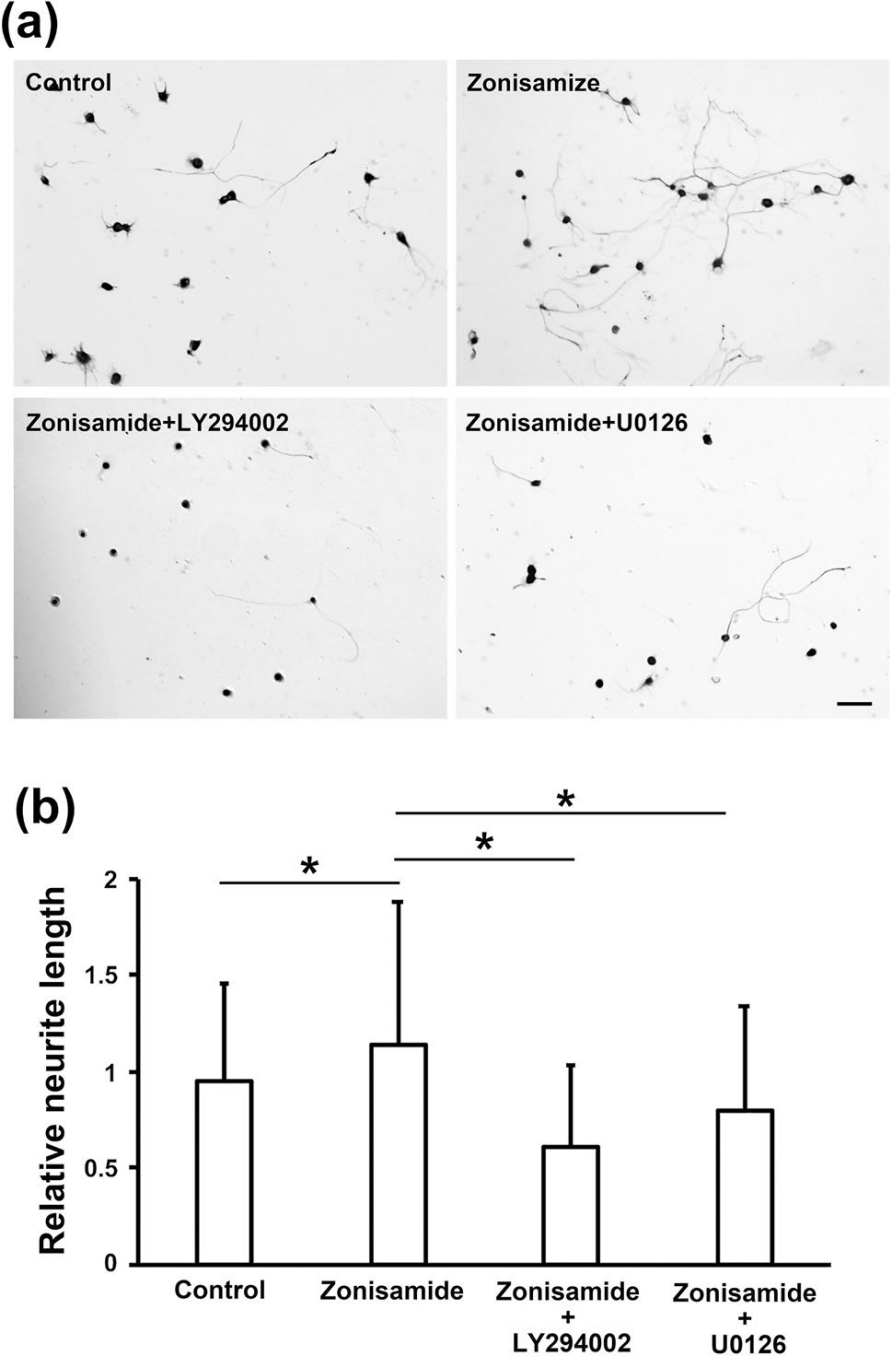
The neurite outgrowth-promoting effects of 100 μM zonisamide are attenuated by co-treatment with 10 μM of LY294002 or U0126. **a** Representative photomicrographs of control, zonisamide-, zonisamide + LY294002-, and zonisamide + U0126-treated DRG neurons immunostained with anti-βIII tubulin antibody. Scale bar = 50 μm. **b** Bar charts of the relative neurite length in each culture condition. Values represent means + SD from 361 to 504 neurites; *P < 0.01

In the previous study (Yagi et al. 2015), zonisamide dose-dependently (1 μM < 10 μM < 20 μM) promoted neurite outgrowth from primary-cultured motor neurons. In agreement with that finding, we observed that treatment with zonisamide enhanced neurite outgrowth from DRG neurons in a concentration-dependent manner; zonisamide at 10 μM and 100 μM significantly increased the relative neurite length as compared with control (Fig. 1). In addition to the neurite length, we measured the diameters of neurons in the control and 100 μM zonisamide-treated groups, respectively, and saw no significant differences in the average value between the former (26.6 ± 2.7 µm; *n* = 90) and the latter (26.1 ± 2.6 µm; *n* = 90). These findings suggest that zonisamide increases the neurite length without altering the size of neuronal cell bodies. Because the promoting effects of zonisamide were significantly attenuated by co-treatment with 10 μM of a PI3K inhibitor LY294002 or a MEK inhibitor U0126 (Fig. 2), PI3K and MAPK signaling pathways appear to play a role in the zonisamide-induced promotion of neurite outgrowth. These findings are similar to those in our previous study (Sango et al. 2008), which described the inhibitory effects of LY294002 and U0126 on the neurite outgrowth-promoting activity of ciliary neurotrophic factor (CNTF). CNTF acts on neurons via the cell-surface receptor complex (CNTF receptor α, gp130, and LIF receptor) to activate the signaling pathways, such as Janus kinase (JAK)/signal transducer and activator of transcription 3 (STAT3), PI3K/AKT, and MEK/MAPK, whereas receptors for zonisamide have not been identified and the mechanisms of its neurotrophic activity remain largely unknown.

**Fig. 3:**
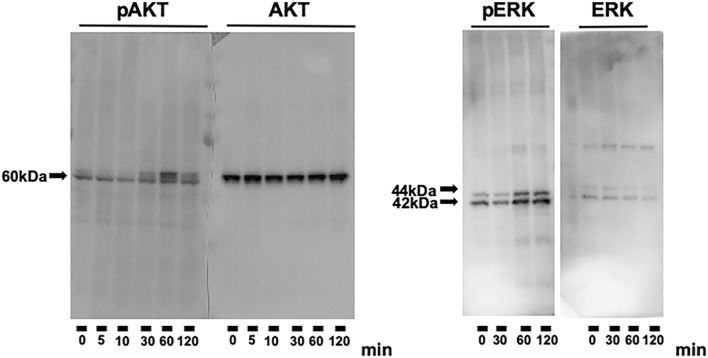
Treatment with 100 μM zonisamide for 60 min or 120 min induces phosphorylation of AKT and ERK1/2 in ND7/23 cells. The representative pictures of the western blot analysis are shown; similar findings are obtained by three experiments

Next, we tried to look into phosphorylation of AKT and ERK1/2 in DRG neurons in the presence or absence of zonisamide; however, the amount of protein obtained from the primary-cultured DRG neurons was insufficient to evaluate the phosphorylated state of each molecule by western blotting. We then substituted them with DRG neuron × neuroblastoma hybrid cells ND7/23, which possess high proliferative activity with some characteristic features of nociceptive sensory neurons, such as expression of substance P and high-affinity neurotrophin receptor TrkA (Wood et al. 1990; Mitani et al. 2016; Yin et al. 2016). The western blot analysis revealed that 100 μM zonisamide for 60 min or 120 min induced phosphorylation of AKT and ERK1/2 in ND7/23 cells (Fig. 3). Numerous kinds of neuroprotective molecules have been shown to enhance neurite outgrowth through the activation of PI3K/AKT and MEK/MAPK pathways (Okada et al. 2010; Zigmond 2012; Chan et al. 2014). In addition, Saijilafu et al. (2013) reported that PI3K pathway activated in response to axonal injury was required for sensory nerve regeneration. Although no direct evidence was provided, PI3K/AKT pathway might be involved in the neuroprotective function of zonisamide. For instance, zonisamide-induced up-regulation of manganese superoxide dismutase through the activation of PI3K pathway might contribute to the attenuation of 1-methyl-4-phenyl-1,2,3,6-tetrahydropyridine (MPTP)-induced apoptotic cell death of SH-SY5Y neuroblastoma cells (Kawajiri et al. 2010). According to the study by Yagi et al. (2015), zonisamide tended to suppress phosphorylation of ERK1/2 in primary-cultured motor neurons and NSC-34 motor neuron-like cells. The reasons for the opposite effects of zonisamide on ERK1/2 phosphorylation between ND7/23 cells and the motor neurons remain unknown, but one of plausible explanations might be the expression of TrkA receptor in the former, but not in the latter. MAPK signal transduction cascade plays a major role in the neurotrophic activity of nerve growth factor (NGF) and related molecules on TrkA-expressing DRG neurons and ND7/23 cells (Ulmann et al. 2009; Inoue et al. 2012), whereas NGF failed to promote the survival of TrkA-negative NSC-34 cells (Matusica et al. 2008). However, it needs further consideration on how zonisamide activates TrkA-MAPK signaling pathway. It is also noteworthy that activation of ERK1/2 in neuronal cells is involved in not only the effects of neuroprotective molecules (Zigmond 2012), but also the negative events, *e.g.* neuronal cell death caused by oxidative stress (Satoh et al. 2000). The findings of our study suggest that zonisamide-induced ERK1/2 phosphorylation contributes to the promotion of neurite outgrowth in DRG neurons, whereas zonisamide might attenuate its phosphorylation to protect motor neurons and NSC-34 cells from oxidative stress-induced injury and death (Yagi et al. 2015). Our current study focuses on the unsolved problems raised above, as well as possible cross talk between the PI3K and MAPK pathways and the downstream targets of these pathways responsible for zonisamide-induced neurite outgrowth.

### Zonisamide does not promote proliferation/survival or migration of IFRS1

**Fig. 4:**
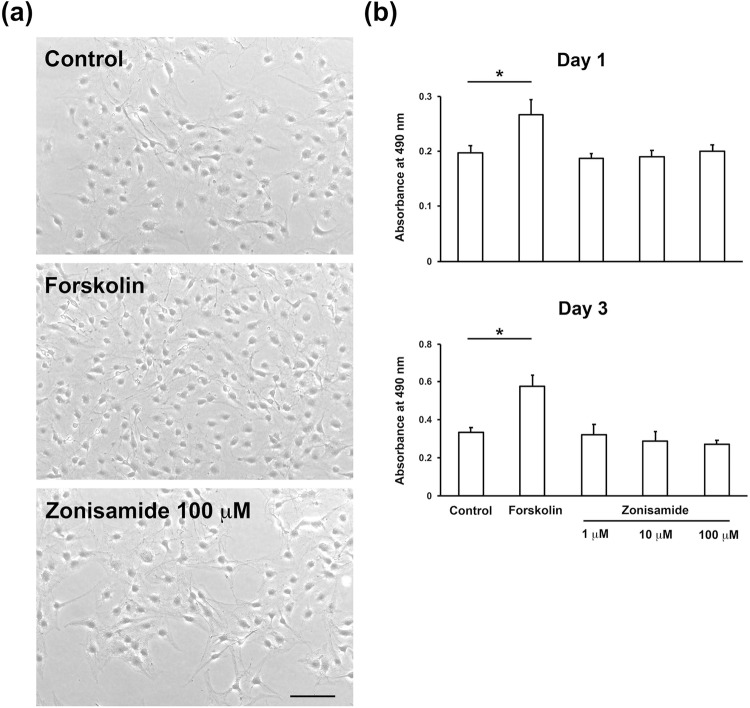
Zonisamide exhibits no significant effects on proliferation/survival of IFRS1; MTS assay. **a** Representative photomicrographs of control, forskolin, and zonisamide 100 μM-treated IFRS1 at Day 1. Scale bar = 100 m. **b** Bar charts of the absorbance at 490 nm at Day 1 and Day 3 after treatment with 2 μM forskolin (a positive control), 1 μM, 10 μM, or 100 μM zonisamide. Values represent means + SD from 7–8 experiments; *P < 0.01

**Fig. 5:**
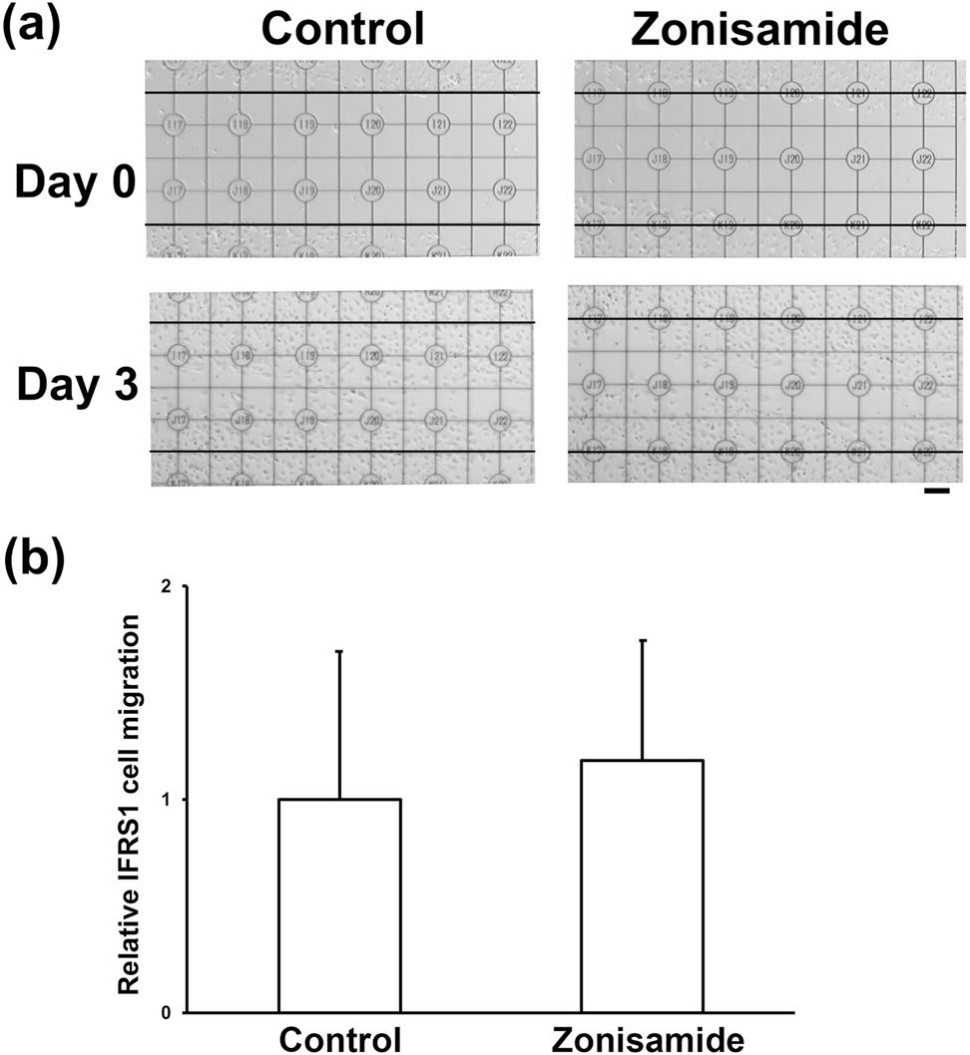
Zonisamide exhibits no significant effects on migration of IFRS1; scratch wound assay. **a** Representative photomicrographs of control and zomisamide (100 μM)-treated IFRS1 at Day 0 (2 h after scratch) and Day 3. Scratch was generated between the 2 thin black lines in each picture. Scale bar = 100 μm. **b** Bar charts of the relative number of migrating cells in the presence or absence of 100 μM zonisamide after 3 days of scratch generation. Values represent means + SD from five experiments

In contrast to its neurite outgrowth-promoting activity described above, zonisamide failed to enhance proliferation/survival (Fig. 4) or migration (Fig. 5) of IFRS1. These findings led us to speculate that zonisamide facilitates axonal regeneration through its direct actions on neurons rather than the stimulation of Schwann cell activity. However, we cannot deny the possibility that zonisamide potentiates synthesis and secretion of neurotrophic factors and cytokines in Schwann cells to augment neuroprotective system against axonal injury. We plan to explore that possibility by employing DNA microarray analysis, real-time RT-PCR analysis, and enzyme immunoassay (Niimi et al. 2018). The findings that zonisamide increased the reduced glutathione (GSH) level in astroglial cells, but not in dopaminergic neurons (Asanuma et al. 2010) suggests its beneficial effects on glial cells to protect the nervous system from oxidative stress and progressive neurodegeneration.

### Potential repositioning of zonisamide for peripheral nerve lesions

The previous (Yagi et al. 2015) and present studies have demonstrated the neurotrophic activity of zonisamide on both motor and sensory neurons in the PNS and suggested its potential utility for peripheral nerve lesions. Because the effective zonisamide concentrations in these studies are 10–100 μM, ranging from those in sera of patients with Parkinson’s disease (up to around 20 μM; Sumitomo Dainippon Pharma Co., Ltd., unpublished data) to those in sera of epilepsy patients (50–200 μM) (Mimaki 1998), it seems fair to suppose that the research protocols have some clinical relevance. However, several limitations of our studies must be considered. First, there is a topographic difference in the site of neurite initiation and elongation; neurites sprout from neuronal cell bodies in vitro, whereas axonal regeneration occurs at the sites of injury in vivo. Second, individual neurons are isolated from other neurons and non-neuronal cells in vitro, and the effects of cellular interplay on axonal regeneration cannot be evaluated. To overcome these defects, we have established a three-dimensional culture system of ganglion explants in which adult peripheral ganglia with nerve fibers are embedded in collagen gel or Matrigel^®^, and neurite outgrowth from transected nerve stump has been evaluated (Horie et al. 1994; Sango et al. 2017). Because of the maintenance of cell-to-cell interactions, it may be reasonable to consider that the explant culture mimics axonal regeneration in vivo better than the use of dissociated cells. Our preliminary study with the explant culture has suggested significant neurite outgrowth-promoting activity of zonisamide, which will be reported elsewhere.

To our knowledge, this is the first study to investigate the bioactivities of zonisamide on DRG neurons and Schwann cells. Although further analyses are needed to elucidate more precise action mechanisms of zonisamide, it is expected that zonisamide will be applicable for accelerating axonal regeneration with functional recovery after peripheral nerve injury and restoring diabetic and other peripheral neuropathies (Hord et al. 2003; Tanabe et al. 2008).
